# Barotrauma and its complications in COVID-19 patients: a retrospective study at tertiary care hospital of Eastern India

**DOI:** 10.1186/s42269-022-00880-3

**Published:** 2022-07-15

**Authors:** Roopak Dubey, Kamal Kumar Sen, Aparajita Mishra

**Affiliations:** 1 Department of Radiodiagnosis, Kalinga Institute of Medical Sciences Bhubaneswar, Bhubaneswar, Odisha India; 2 Department of Community Medicine, Kalinga Institute of Medical Sciences Bhubaneswar, Bhubaneswar, Odisha India

**Keywords:** Pneumothorax, Pneumomediastinum, Subcutaneous emphysema, Respiratory distress syndrome, COVID-19 cases

## Abstract

**Background:**

The development of barotrauma in COVID-19 patients who were ventilated and admitted to the intensive treatment unit seemed to have been a problematic issue in the COVID era. This study aimed to explore the possibility of developing the barotrauma-related issues with mechanical ventilation in the cases of individuals suffering from COVID-19.

**Results:**

Out of 48 patients who developed barotrauma, 30 (62.5%) presented with pneumothorax, 22 (45.8%) with pneumomediastinum, 10 (20.8%) with subcutaneous emphysema, and 2 (4.1%) with pneumopericardium. Of those that developed barotrauma, 45 (93.7%) patients were in acute respiratory distress syndrome. In patients with and without barotrauma, significant factors were white blood cell count (*p* = 0.001), neutrophil percentage (*p* = 0.012), and lymphocyte percentage (*p* = 0.014). There were no statistically significant differences in CRP, procalcitonin, d-dimer test, LDH, or ferritin.

**Conclusions:**

Patients infected with COVID-19 have a high risk of barotrauma when on mechanical ventilation. As a result, the death rate in this patient group is higher.

## Background

COVID-19, a novel coronavirus, has spread around the world (World Health Organization 2. WHO Director-General’s remarks at the media briefing 2020). This highly transmissible and contagious disease affects a variety of systems, including the respiratory tract. In COVID-19 patients in the intensive care unit (ICU), pneumothorax is reported to occur at a rate of 2% (World Health Organization 2. WHO Director-General’s remarks at the media briefing 2020; Chan et al. 2020 Feb 15). Recent studies have found that barotrauma-related issues caused by invasive mechanical ventilation are becoming increasingly prevalent, with up to 15% of COVID-19 patients experiencing them (Bajema et al. 2020 Feb 14). In mechanically ventilated patients, the relationship between acute respiratory distress syndrome (ARDS) and subsequent pneumothorax has long been recognised as a risk factor for fatality (Huang et al. 2020 Feb 15; Chen et al. 2020 Feb 15; Wang et al. 2020 Mar 17).

Patients with ARDS and COVID can benefit from positive pressure ventilation (PPV), which is a non-physiological and invasive/non-invasive approach that can save their lives. It has its own set of hazards and consequences, including pulmonary barotrauma (PBT) and ventilator-induced lung injury, both of which have been related to multisystem organ failure in ARDS patients (Slutsky and Ranieri 2013 Nov 28).

Using a low tidal volume (Vt) and a high positive end-expiratory pressure (PEEP) in patients with ARDS has been shown in several trials to minimise hospital mortality (Walkey et al. 2017). Low Vt (4–8 ml/kg) is suggested by numerous standards, including the COVID-19 guidelines from the last surviving sepsis campaign, since it is considered to decrease volutrauma in ARDS patients' lungs (Alhazzani et al. 2021 Mar 1; Goligher et al. 2017; Levy et al. 2018 Jun). Limiting lung damage to plateau pressures (Pplat) of less than 30 cm H2O is another lung-protective strategy, since Pplat > 32 has been associated with higher short-term mortality (Yasuda et al. 2017 May 1). During the COVID pandemic, extrinsic PEEP has been utilised in ARDS to improve oxygenation, lower oxygen demand (FiO2), and minimise atelectotrauma (repeated opening and closing of alveoli), all of which helped to prevent ventilator-induced lung damage (Alhazzani et al. 2021 Mar 1). In trials, higher PEEP techniques have been shown to improve survival in ARDS patients at the expense of an increased risk of pneumothorax (Briel et al. 2010 Mar 3). To ensure adequate oxygenation, PEEP might be raised at the risk of increased Pplat and barotrauma.

In mechanical ventilation, PBT refers to alveolar rupture caused by increased transalveolar pressure (the alveolar pressure minus the pressure in the adjacent interstitial space), which causes air leaks into extra-alveolar tissue, resulting in pneumothorax, pneumomediastinum, pneumoperitoneum, and subcutaneous emphysema (Udi et al. 2021 Apr). These patients frequently require a high level of PEEP to maintain oxygenation, putting them at risk for barotrauma.

The objective of our study was to identify clinical features and risk factors associated with the development of barotrauma in COVID-19 patients who were mechanically ventilated. The goal of the study was to see how COVID-19 affects the clinical course and prognosis of barotrauma-related ailments.

## Methods

### Design of the study and data gathering

Between March 2021 and June 2021, a retrospective research was conducted at the Kalinga Institute of Medical Sciences in India. The hospital's institutional ethics committee granted clearance for the project. The ethics committee approved a waiver of consent from individual patients due to the retrospective nature of the study.

A total of 350 patients were evaluated after reverse transcriptase polymerase chain reaction (RTPCR) nasal swab tests revealed that they had COVID-19 infection and were sent to the hospital's intensive care unit (ICU) for mechanical ventilation. All patients who developed PBT (pneumothorax, pneumomediastinum, pneumopericardium, or subcutaneous emphysema) and received any form of PPV, including non-invasive positive pressure ventilation (NIPPV) such as continuous positive airway pressure (CPAP) or bi-level positive airway pressure (BiPAP) and those who required invasive positive pressure ventilation (IPPV), were included.

Individuals having a pre-existing pneumothorax at the time of admission, as well as those with identified iatrogenic pneumothorax episodes, were excluded.

Clinical data were obtained retrospectively by looking through the medical records of the patients. The hospital database was used to get information such as age, sex, chronic medical conditions, and history of underlying lung disease, as well as presenting symptoms, laboratory values on admission, and outcomes (death or discharge).

### Ventilation protocol

All IPPV patients were treated with lung-protective ventilation strategies that included a low tidal volume (Vt) of 4–8 mL/kg predicted body weight, a volume-limited assist control mode, and an initial positive end-expiratory pressure (PEEP) of 5 cm H2O that was gradually increased to improve oxygenation while aiming for a plateau pressure (Pplat) of 30 cm H2O to avoid lung injury. According to our hospital's sedative and muscle relaxant protocol, benzo-diazepines infusions were used in patients with ARDS in combination with a powerful analgesic such as fentanyl or morphine. Patients who were getting IPPV should follow this sedative regimen. Patients who received NIPPV, on the other hand, were not given sedatives or analgesics and were only given minimal dosages as needed for comfort.

### Data analysis

HRCT chest imaging/X-rays were used to radiologically identify PBT. An ICU posted radiologist analysed and evaluated HRCT data and chest X-rays.

All of the data were examined using SPSS (IBM, version 25). For continuous data, descriptive statistics were used to calculate mean and standard deviations, whereas frequency statistics were employed to create numbers and percentages for categorical variables. The statistical significance was determined using a p value of less than 5%.

## Results

**Fig. 1:**
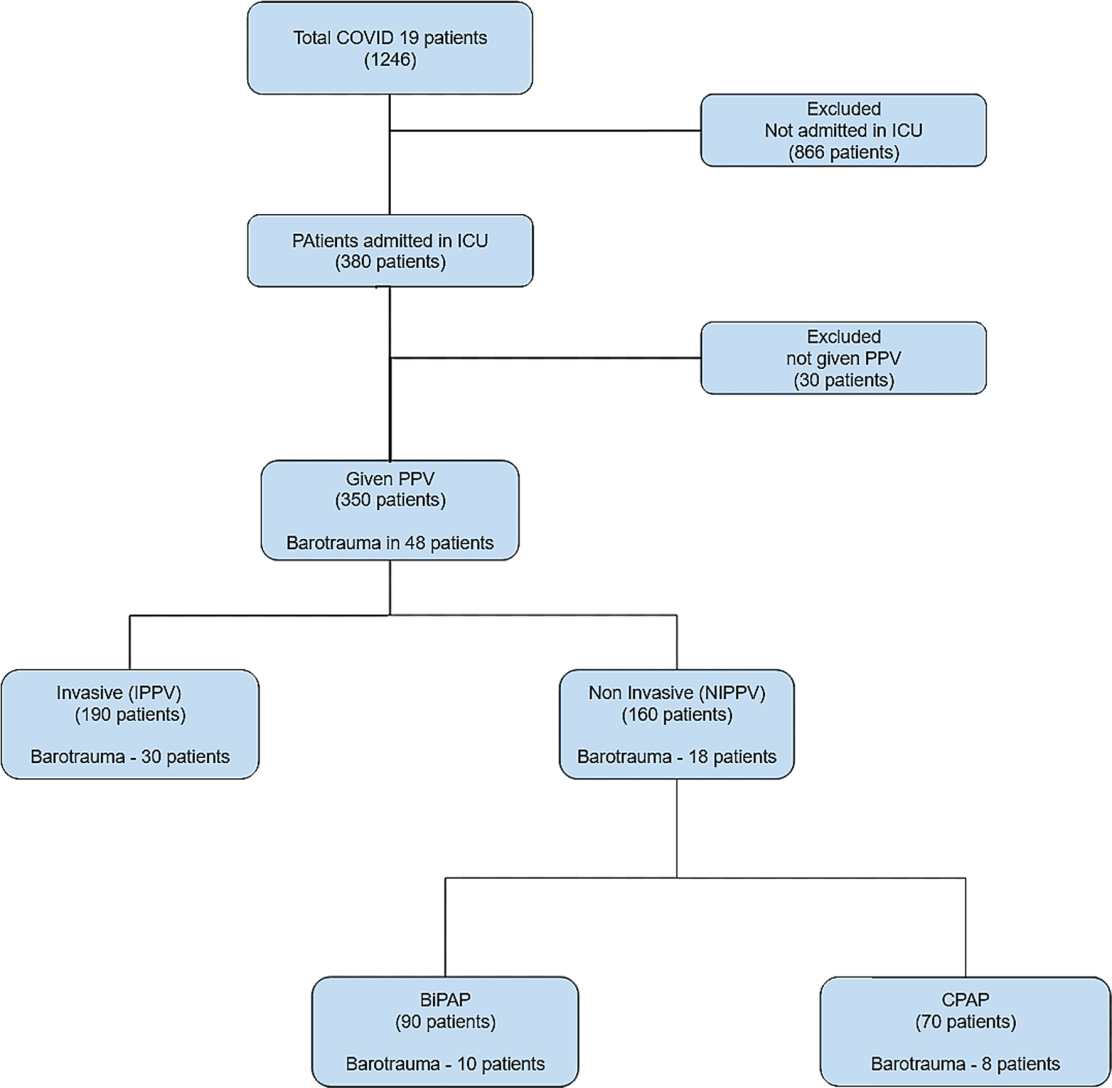
Distribution of patients admitted in our hospital from March to June 2021

The 350 patients in the study had an average age of 55.9 (13.5) years, with 270 (77.14%) of them being male. Data on demographics, clinical care, and biochemistry are shown in Table 1. Throughout their hospital stay, all PPV patients were divided into two groups. The first group included 48/350 (13.7 percent) individuals who acquired PBT after receiving any form of PPV. The second group consisted of 302 individuals (86.28%) who had received any type of PPV but had not acquired PBT. Out of 350 patients who received PPV, 190 patients (54.28%) received IPPV, and 30 patients developed PBT. On the other hand, NIPPV was given to 160 patients (45.71 percent). Ninety patients got BiPAP, and 70 patients received CPAP, with 10 and 8 patients developing PBT, respectively, after receiving NIPPV (Fig. 1).

**Table 1:** Demographic, clinical, and biochemical characteristics according patients with or without barotrauma

Characteristics	Barotrauma (*n* = 48)	No barotrauma (*n* = 310)	*p* value^*^
*Demography*			
Age in years (SD)	55.9 (13.5)	56.0 (13.3)	0.12
Males	38 (79.1%)	220 (70.9%)	0.23
Females	10 (20.9%)	46 (29.1%)	0.45
*Symptoms*			
Shortness of breath	30 (62.5%)	210 (67.7%)	0.75
Cough	24 (50.0%)	118 (38.0%)	0.34
Sore throat	10 (20.8%)	78 (25.1%)	0.56
Hemoptysis	5 (10.4%)	43 (13.8%)	0.12
Unconsciousness	26 (54.1%)	190 (61.2%)	**0.01**
*Comorbidities*			
Diabetes	10 (20.8%)	60 (19.3%)	0.08
Hypertension	9 (16.6%)	54 (17.5%)	0.12
Asthma	7 (12.5%)	34 (11.0%)	0.23
COPD	3 (6.2%)	20 (6.4%)	0.44
CKD	2 (4.1%)	16 (5.1%)	0.09
*Biochemical*			
WBC (SD)	12.7 (7.7)	8.5 (4.8)	**0.001**
Neutrophils (SD)	84.8 (12.4)	78.0 (12.9)	**0.012**
Lymphocytes (SD)	9.8 (10.5)	13.8 (10.1)	**0.014**
CRP (SD)	134.0 (111.4)	142 .4 (119.6)	0.23
Procalcitonin (SD)	4.3 (12.3)	22.2 (242.7)	0.10
D-dimer assay (SD)	2216.2 (3918.1)	2565.6 (3191.5)	0.32
LDH (SD)	1005.9 (3513.6)	732.0 (270.2)	0.10
Ferritin (SD)	1413.8 (1966.4)	1082.8 (1490.1)	0.23
Length of Stay, days (SD)	16.4 (8.5)	20.1 (14.0)	0.12
*Pressure*			
PEEP minimum (SD)	7.7 (2.6)	6.7 (3.8)	0.08
PEEP Maximum (SD)	14.5 (2.7)	12.5 (5.7)	**0.002**
*Outcome*			
Deaths	35/48 (72.9%)	120/302 (39.7%)	**0.015**
Discharged	13/48 (27.1%)	182/302 (60.2%)	0.08

Unless otherwise stated, data for continuous variables are provided as means (standard deviations). For categorical variables, data are given as a number (%). The *significance level was set at p value less than 0.05. Significant *p* values have been shown in bold numericals

**Fig. 2:**
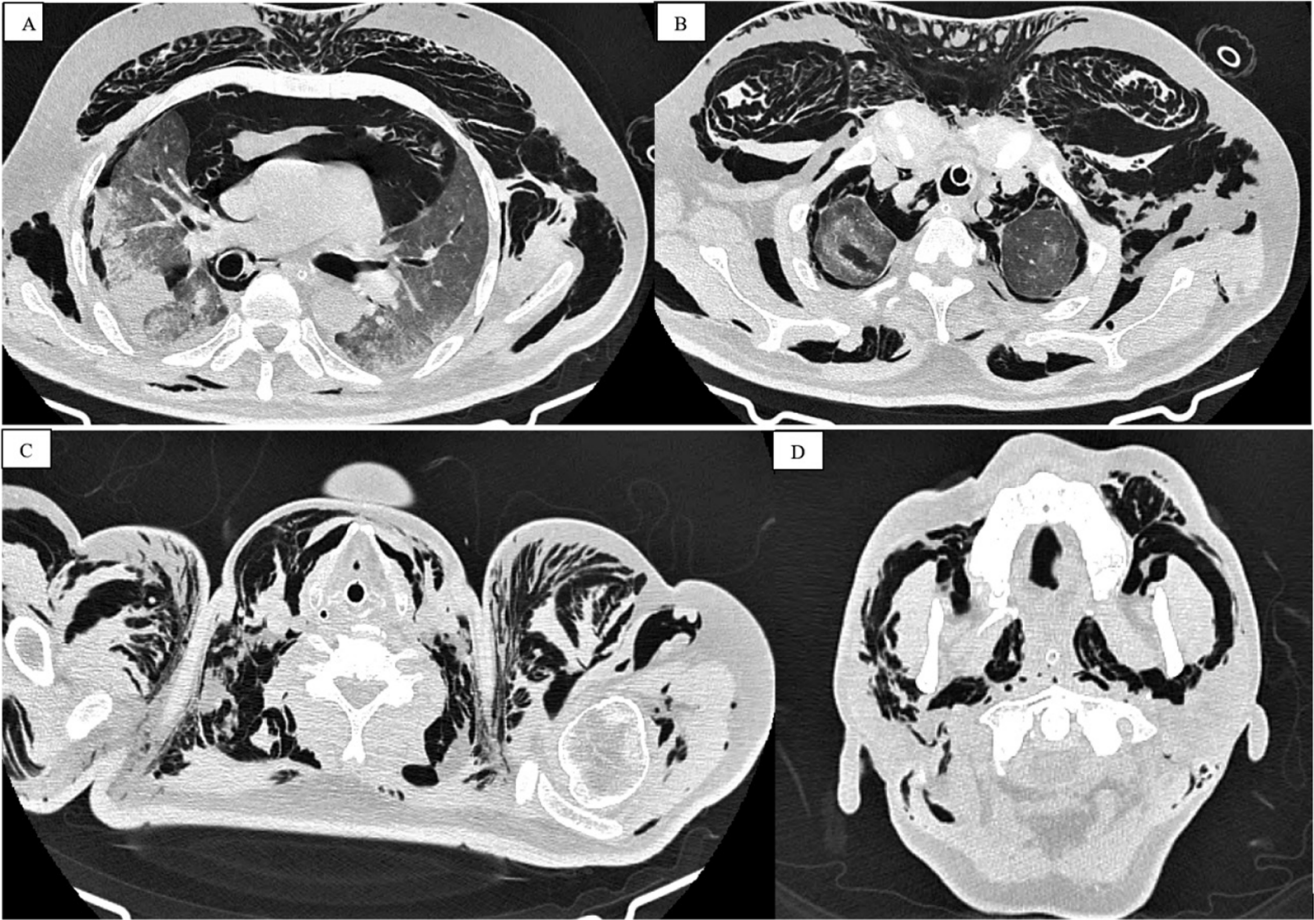
A 56-year-old male was brought to our hospital with worsening shortness of breath. The patient was moved to the critical care unit after failing to maintain a SpO2 of over 87 percent on the fourth day of hospitalisation (ICU). Within 3 days of intubation, axial CT thorax sections indicated severe barotrauma in the form of pneumomediastinum, pneumothorax, and subcutaneous emphysema (**A**, **B** and **C**). Subcutaneous emphysema had also spread to the neck and face (**D**)

**Fig. 3:**
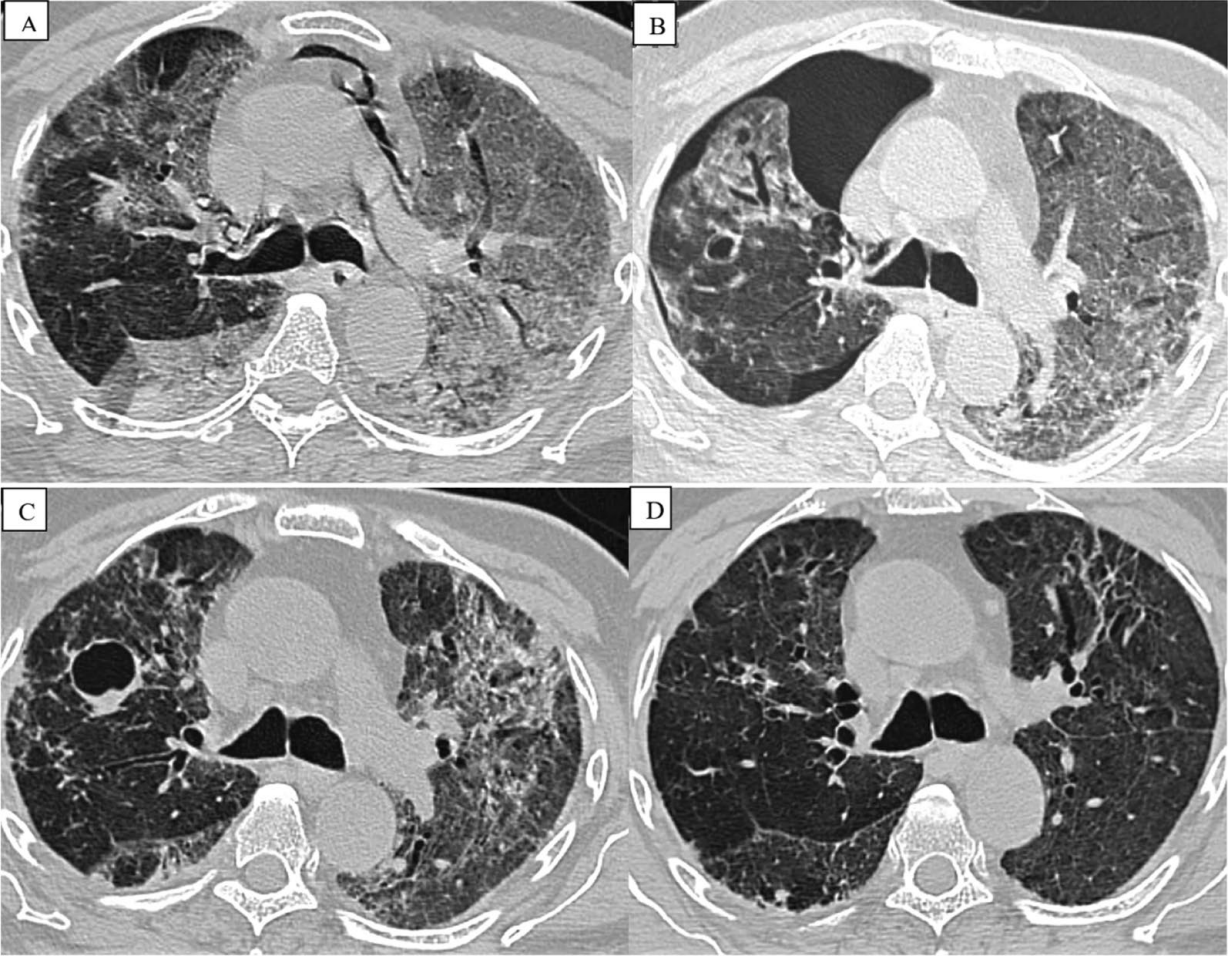
A 62-year-old COVID-positive man with acute dyspnea and cough was brought to the hospital. The patient was suffering from acute ARDS. He was admitted to the intensive care unit and placed on mechanical ventilation. The first HRCT scan **A** indicated significant consolidation and ground glass opacities in both lungs with pneumomediastinum. He developed pneumothorax on repeat CT **B** after 7 days of mechanical ventilation. The patient's clinical state improved over time, and a 20-day CT scan **C** revealed complete resolution of the pneumothorax and pneumomediastinum, but a new cavity had developed in the anterior portion of the right lung. Patient followed up after 3 months **D** revealed complete resolution of cavity with mild residual fibrosis in both lungs

Out of 48 patients who developed barotrauma, 30 (62.5%) presented with pneumothorax, 22 (45.8%) with pneumomediastinum, 10 (20.8%) with subcutaneous emphysema, and 2 (4.1%) with pneumopericardium (Table 2) (Figs. 2 and 3).

**Table 2:** Types of barotrauma and categories of ACUTE respiratory distress syndrome

Types of barotrauma (n = 48)	
Pneumothorax	30 (62.5%)
Pneumomediastinum	22 (45.8%)
Subcutaneous emphysema	10 (20.8%)
pneumopericardium	2 (4.1%)
Acute respiratory distress syndrome (ARDS) (n = 45/48)	
Mild	30/48 (62.5%)
Moderate	10/48 (20.8%)
Severe	5/48 (10.4%)

In our study, 45 (93.7%) of the total patients (*n* = 48) were suffering from ARDS. At the time of the barotrauma, 30/48 (62.5%) of these patients had severe ARDS (PaO2/ FiO2 of 100), whereas 10/48 (20.83) had moderate ARDS (PaO2/ FiO2 of 100–200) (Table 2).

Several inflammatory markers were shown to be statistically different between people who had barotrauma and those who did not. Significant factors were white blood cell count (WBC) (*p* = 0.001), neutrophil percentage (*p* = 0.012), and lymphocyte percentage (*p* = 0.014) (Table 1). There were no statistically significant differences in CRP, procalcitonin, d-dimer test, lactate dehydrogenase (LDH), or ferritin.

All 48 barotrauma patients were mechanically ventilated. Mortality was substantially higher among individuals with barotrauma 35/48 (72.9%) compared to those without barotrauma 120/302 (39.73%) (*p* = 0.015). The maximal PEEP in barotrauma patients was significantly greater than in non-barotrauma patients (*p* = 0.002). There were no significant changes in demographic parameters or length of hospital/ICU stay between patients with and without barotrauma. Despite the fact that the prevalence of comorbidities was not statistically significant in patients with and without barotrauma (*p* = 0.57), the majority of PPV patients had underlying comorbidities, the most common of which were hypertension (HTN) and diabetes mellitus (DM). All of the patients on mechanical ventilation were managed with lung-protective measures. A tube thoracostomy was placed at the bedside of patients who suffered a pneumothorax.

The characteristics of patients who received IPPV versus NIPPV were compared in a sub-analysis. IPPV-treated patients had a higher death rate (*p* = 0.003).

Six people in our research showed symptoms of barotrauma before starting mechanical breathing (findings of pneumomediastinum, subcutaneous emphysema, or both). To maintain track of these people, clinical examinations and serial chest X-rays were employed.

**Fig. 4:**
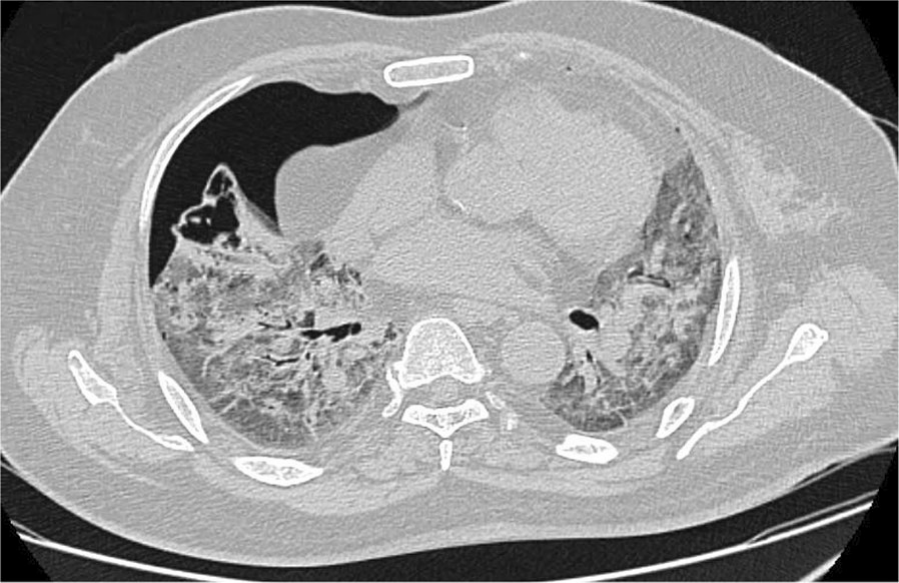
Axial HRCT scan of a 52-year-old male revealed pneumothorax on the right side. A cavitary lesion with irregular margins and few internal septations was detected in the right middle lobe, indicating the likelihood of a fungal infection within the cavity. A culture test revealed that the patient had candidiasis. Mechanical ventilation was the cause of the pneumothorax. Cavity formation might be caused by invasive candidiasis, mechanical ventilation, or a combination of the two

Besides established complications like pneumothorax, pneumomediastinum, and subcutaneous emphysema, few other findings like cystic bronchiectasis and cavitary lesions were also seen in patients admitted in ICU with PPV. Three of the 48 patients had fungal development in their pre-existing cavities. Two of them were attributable to candida species, while one was due to mucor infection, according to fungal culture test (Fig. 4).

## Discussion

The present study focused at COVID-19 patients and looked at risk factors for barotrauma as well as clinical consequences for management. COVID-19 patients suffer from barotrauma, which has been thoroughly documented in the literature (Abdallat et al. 2020; McGuinness et al. 2020 Nov). In our single-centre investigation, the incidence of barotrauma-related issues in COVID-19 patients was 13.7 percent, which is virtually identical to the rate described by McGuinness G et al. (McGuinness et al. 2020 Nov).

Pre-existing lung disease and the development of barotrauma during invasive ventilation administration have been highlighted in several additional reports. Pneumonia, chronic obstructive pulmonary disease (COPD), and lung cancer, in particular, have all been shown to increase the risk of barotrauma (McGuinness et al. 2020 Nov). Similarly, all of the patients with barotrauma in the current research (*n* = 48) had COVID-19 pneumonia, 12.5 percent had asthma, and 6.2 percent had a history of COPD.

Total 350 patients were provided mechanical ventilation in our research, and 13.7 percent (*n* = 48) of them had barotrauma, which was radiologically confirmed as a pneumothorax, pneumomediastinum, or surgical emphysema. PBT was found in 10–14 percent of COVID-19-infected mechanically ventilated ARDS patients in many case series researches, which mirrored our findings (McGuinness et al. 2020 Nov; Lemmers et al. 2020; Capaccione et al. 2021 Feb; Edwards et al. 2021 Jan 1). Martinelli et al. found that around 25% of intubated patients acquired pneumothorax or pneumomediastinum, which is higher than the incidence (13.7%) identified in our group (Martinelli et al. 2020).

In terms of biochemical results, we discovered that patients who experienced barotrauma had higher WBC, neutrophil counts, and LDH. These findings are comparable to those of Shioe et al., who observed that patients infected with the SARS-CoV virus had greater levels of LDH and neutrophils, which were likewise linked to a higher degree of lung damage. However, in that study, the findings were not compared to those of individuals who did not experience barotrauma (Sihoe et al. 2004 Jun 1).

When invasive ventilation is utilised, ARDS has been established as an independent risk factor for barotrauma. In the literature, the incidence of ARDS has been reported to fluctuate, with some studies showing rates as high as 15% (Gammon et al. 1992 Aug 1; Eisner et al. 2002; Gammon et al. 1995 Oct; Weg et al. 1998 Feb 5; Acute Respiratory Distress Syndrome Network 2000 May 4). In our study, 45 (93.75%) of the 48 patients who had barotrauma satisfied the ARDS criterion.

Furthermore, in our study, patients who had barotrauma had a higher maximum PEEP than patients who did not develop barotrauma (*p* = 0.002). High PEEP has been frequently employed as part of a ventilator management approach. This has been linked to barotrauma, which necessitates thoracostomy drainage in certain cases. This condition can induce serious hemodynamic alterations and is linked to a greater death risk in these individuals.

To decrease barotraumatic occurrences in COVID-19, low tidal volumes (4–8 ml/kg) and plateau pressures (Pplat) of less than 30 cm H2O should be used in practise. However, in some cases, a patient may require greater PEEP to achieve adequate oxygenation, which may raise the risk of higher Pplat and barotrauma. As a result, when more PEEP is administered, doctors must calculate the risk vs. benefit ratio.

High PEEP, high tidal volumes, and high plateau pressures are the main variables which increase the occurrence of barotraumatic events.

In our study, six people had spontaneous pneumomediastinum or pneumothorax. Similar case reports from China described spontaneous pneumomediastinum in COVID-19 patients (Eisner et al. 2002). Before starting mechanical ventilation, these patients experienced spontaneous pneumomediastinum or pneumothorax, which might suggest a severe kind of lung injury associated with COVID-19 infection.

Three patients with pneumothorax and/or pneumomediastinum were found to have cavitary lesions in the lung parenchyma, which demonstrated fungal growth on culture. These cavities might be pre-existing as a result of PPV-induced barotrauma, or they could be developed by invasive fungal infections on their own. But definitely, mechanical ventilation could be a contributing factor in the development of these cavities, which could be exacerbated by fungus infection. The radiologists play an important role in detecting barotraumatic events (like pneumothorax, pneumomediastinum, subcutaneous emphysema, and pneumopericardium) in COVID patients as these patients undergo HRCT or X-ray chest at least once in their course of disease.

In line with the findings of De Lassence et al., our investigation found that patients who acquired barotrauma had a substantially higher death rate (72.9%) than patients (39.73%) who did not develop barotrauma (*p* = 0.015) (Lassence et al. 2006 Jan 1). It is worth noting that the mortality rate was also substantial among patients who did not have barotrauma-related events; this might reflect a morbid cohort of ICU patients.

## Conclusions

In COVID-19 patients who are mechanically ventilated, there is a substantial risk of barotrauma. COVID-19 is, in fact, a risk factor for the development of spontaneous pneumothorax and/or pneumomediastinum, as demonstrated by the presence of these conditions in 6 of our sample members. In our patient sample, we discovered many variables that have been associated with the development of barotrauma-related sequelae. It is a delicate balancing act to find the optimal ventilator settings for lung recruitment while avoiding barotrauma. COVID-19 patients who had barotrauma had a significant mortality rate, according to our findings. The role of radiologist is important in the early detection of barotrauma and may be able to aid in the treatment of its complications.

## Data Availability

The datasets generated and/or analysed during the current study are not publicly available but are available from the corresponding author on reasonable request.

## Abbreviations

ARDS: Acute respiratory distress syndrome
ICU: Intensive care unit
PPV: Positive pressure ventilation
PBT: Pulmonary barotrauma
Vt: Tidal volume
PEEP: Positive end-expiratory pressure
Pplat: Plateau pressures
RTPCR: Reverse transcriptase polymerase chain reaction
NIPPV: Non-invasive positive pressure ventilation
CPAP: Continuous positive airway pressure
BiPAP: Bi-level positive airway pressure
IPPV: Invasive positive pressure ventilation
DM: Diabetes mellitus
HTN: Hypertension
COPD: Chronic obstructive pulmonary disease
WBC: White blood counts
LDH: Lactate dehydrogenase
